# Patient Characteristics and Comorbidities Influence Walking Distances in Symptomatic Peripheral Arterial Disease: A Large One-Year Physiotherapy Cohort Study

**DOI:** 10.1371/journal.pone.0146828

**Published:** 2016-01-11

**Authors:** Sarah Dörenkamp, Ilse Mesters, Rob de Bie, Joep Teijink, Gerard van Breukelen

**Affiliations:** 1 Department of Epidemiology, Functioning and Rehabilitation Programme, CAPRHI School for Public Health and Primary Care, Maastricht University, Maastricht, The Netherlands; 2 Department of Vascular Surgery, Catharina Hospital, Eindhoven, The Netherlands; 3 Department of Methodology and Statistics, CAPRHI School for Public Health and Primary Care, Maastricht University, Maastricht, The Netherlands; University of Catania, ITALY

## Abstract

**Objectives:**

The aim of this study is to investigate the association between age, gender, body-mass index, smoking behavior, orthopedic comorbidity, neurologic comorbidity, cardiac comorbidity, vascular comorbidity, pulmonic comorbidity, internal comorbidity and Initial Claudication Distance during and after Supervised Exercise Therapy at 1, 3, 6 and 12 months in a large sample of patients with Intermittent Claudication.

**Methods:**

Data was prospectively collected in standard physiotherapy care. Patients received Supervised Exercise Therapy according to the guideline Intermittent Claudication of the Royal Dutch Society for Physiotherapy. Three-level mixed linear regression analysis was carried out to analyze the association between patient characteristics, comorbidities and Initial Claudication Distance at 1, 3, 6 and 12 months.

**Results:**

Data from 2995 patients was analyzed. Results showed that being female, advanced age and a high body-mass index were associated with lower Initial Claudication Distance at all-time points (p = 0.000). Besides, a negative association between cardiac comorbidity and Initial Claudication Distance was revealed (p = 0.011). The interaction time by age, time by body-mass index and time by vascular comorbidity were significantly associated with Initial Claudication Distance (p≤ 0.05). Per year increase in age (range: 33–93 years), the reduction in Initial Claudication Distance was 8m after 12 months of Supervised Exercise Therapy. One unit increase in body-mass index (range: 16–44 kg/m^2^) led to 10m less improvement in Initial Claudication Distance after 12 months and for vascular comorbidity the reduction in improvement was 85m after 12 months.

**Conclusions:**

This study reveals that females, patients at advanced age, patients with a high body-mass index and cardiac comorbidity are more likely to show less improvement in Initial Claudication Distances (ICD) after 1, 3, 6 and 12 months of Supervised Exercise Therapy. Further research should elucidate treatment adaptations that optimize treatment outcomes for these subgroups.

## Introduction

Identifying subgroups of patients who benefit less from physiotherapy treatment is a crucial preparatory step to be able to early adjust treatment expectation and treatment programme. The primary treatment modality for patients with Peripheral Arterial Disease (PAD) is physiotherapy and practice results show a high variety in treatment outcome.

Peripheral Arterial Disease in the leg(s) is a manifestation of narrowing or blockage of arteries (atherosclerosis) that causes poor blood flow to the lower extremities. The clinical spectrum of patients ranges from asymptomatic atherosclerosis to those with the common symptoms of Intermittent Claudication (IC), e.g. tight, aching, or squeezing pain in the calf, thigh or buttock. Symptoms occur when the delivery of oxygen is inadequate to meet the metabolic requirements of the active skeletal muscle [1]. Leg pain is relieved by rest. Intermittent Claudication leads to a moderate or severe impairment in walking ability. In order to improve walking ability, Supervised Exercise Therapy (SET) by a physical therapist is recommended as the primary training modality for this patient group [2–4]. Walking limitation is measured by the Initial Claudication Distance (ICD), the distance at which the patient experiences the onset of claudication pain and the Absolute Claudication Distance (ACD), the distance at which the pain forces the patient to stop walking [3].

A few small scale studies indicate that patient characteristics and comorbid conditions might lead to considerably poorer treatment outcomes during and after SET [5–10]. For example, Katzel et al. [6] (*N* = 141) pointed out that current smokers had a shorter time to onset of claudication pain compared to former or never smokers (188± 28 s vs. 173±25 s and 309± 54 s, respectively; p = 0.023). A reduced improvement after conservative treatment by risk factor modification and physical training for female patients (hazard ratio (HR) 0.51; p = 0.052) was presented by Amighi et al. [7] (*N* = 181). Dolan et al. [8] (*N* = 460) observed that after adjusting for age, PAD patients with diabetes type 2 had a shorter mean 6-min walking distance (1,040 vs. 1,168 feet; p<0.001). A recent study of Farah et al. [9] (*N* = 415) explored that Cardiovascular Disease (CVD) was associated with lower ACD and ICD (OR = 2.45; CI:1.11–5.39 and OR = 1.88; CI:0.86–4.09, respectively) and age, body-mass index (BMI), pulmonary disease and smoking behavior were predictive variables for ACD that were presented by Kruidenier et al. [10]. Taken together, from a scientific point of view these studies seem to provide compelling evidence that specific patient characteristics and comorbidity influence physiotherapy treatment success in patients with IC. However, all previous investigations were based on small numbers of patients (maximum *N* = 460 [7]) largely recruited in the hospital setting. By the use of our large cohort we want to check whether the hampering effects of patient-related variables and comorbidity remain in place in a large population of patients with IC that follow SET in the usual care setting. Moreover, the large sample size of our cohort makes it possible to use a multilevel model through which it can be acknowledged that patients received treatment by different physiotherapists [11].

If the results of our large physiotherapy cohort show that the inverse association between patient characteristics and comorbidity (as identified in earlier small scale studies) remains, subgroups of patients that benefit less from SET can most certainly be identified. The aim of this study is to investigate the association between age, gender, BMI, smoking behavior, orthopedic comorbidity, neurologic comorbidity, cardiac comorbidity, vascular comorbidity, pulmonic comorbidity, internal comorbidity and initial claudication distance (ICD) during and after supervised treadmill exercise at 1, 3, 6 and 12 months in a large physiotherapy cohort study.

## Methods

### Study design and setting

To study the association between patient characteristics and comorbidity on Initial Claudication Distance (ICD) analyses on prospectively collected Electronic Medical Record (EMR) data was performed. Registration of the patients’ medical data in the EMR was part of standard physiotherapy care. Data from patients with IC that were treated with community-based supervised exercise therapy (SET) between 2006 and 2011 was used for this study. The ethics committee/institutional review board 'Medisch Ethische Toetsingscommissie van Atrium Medisch Centrum, Orbis Medisch en Zorgconcern en Zuyd Hogeschool' approved at August, 19th 2013 the present physiotherapy cohort study. Data was gathered in the context of usual care. All participants signed written informed consent for their clinical records to be used in the present physiotherapy cohort study and patient records/information was anonymized and de-identified prior to analysis (METC number: 13-N-85). To enhance transparency this article is written according to the STROBE checklist for cohort studies.

### Participants

All patients with a physician confirmed IC who started community-based SET were eligible for inclusion. The diagnosis of IC was confirmed with an ankle-brachial index (ABI) <0.9 at rest. Exclusion criteria were the absence of a baseline measurement, less than one valid ICD measurement and ICD of 1600 meters or more at baseline [12]. A cut-off value for all ICD was set at 1600m. For practical reasons incline and testing duration in standard practice are maximized to 30 minutes (1600m).

### Variables

The primary outcome measurement was ICD at 1, 3, 6 or 12 months of SET. In recent years, there has been an increased focus on patient-centered care, defined by the Institute of Medicine (IOM) as ‘providing care that is respectful of and responsive to individual patient preferences, needs, and values, and ensuring that patient values guide all clinical decisions’ [13]. The hindrance of patients with IC is that they are limited by pain while walking. During physiotherapy treatment patients are instructed to walk as far as they can through the pain (ACD), until claudication pain becomes so severe that the patient is forced to stop [4]. However, the ACD does not correspond with the distance a patient would walk in the course of daily activities as few will by choice walk until their maximum pain threshold. To acknowledge the importance of the patients’ wish to walk longer distances pain-free this study focuses on Initial Claudication Distance (ICD) as primary outcome to reveal determinants of walking distance in standard physiotherapy care. Independent variables include age, gender and the physiotherapy practice. Besides, BMI of each patient was calculated as weight (kg)/height squared (m^2^) according to the clinical guideline on the Identification, Evaluation and Treatment of Overweight and Obesity in Adults [14]. Smoking behaviour was recorded as non-smoker, previous smoker and current smoker. Presence or absence of orthopaedic-, neurologic-, cardiac-, pulmonic- and internal comorbidity was registered according to the standard hospital registration. Besides, the presence or absence of vascular comorbidity was registered in the same way. As patients with IC already suffer from a vascular disease in the category vascular comorbidity solely the presence or absence of Percutaneous Transluminal Angioplasty (PTA), recanalization, bypass operation, endarterectomy, hypertension, arrhythmia, cardiac decompensation and heart valve suffering was recorded. Detailed information about specific diseases that belong to the other mentioned five categories of comorbidity is reported in S1 File. Patient-related variables and comorbidity were evaluated at baseline.

### Data source

Data for this cohort study was derived from prospectively collected data filled in by physiotherapists in electronic medical records as part of standard care. This registration tool was commissioned by the centre of evidence-based physiotherapy (www.cebp.nl) and realised by Convenience-Fastguide® (www.fastguide.eu). All patients provided their informed consent to register and use their medical data.

### Supervised Exercise Therapy

All patients received SET according to the evidence-based IC guideline of the Royal Dutch Society for Physiotherapy [12] as part of usual care. A standardised graded treadmill test with a constant speed of 3.2 km/h according to Gardner et al. [15] was used as measure of ICD. The incline starts at 0% and is increased by 2% every two minutes. The ICD was evaluated by the physiotherapist at baseline and after 1, 3, 6 and 12 months of follow-up. All measurements were registered in the EMR by the physiotherapist. Detailed information on the community-based exercise program has previously been published by our research group [16,17].

### Statistical methods

Data was analysed with a three-level mixed linear regression analysis. The three levels were physiotherapist, patients within therapists, and measurements within patients. Time and patient characteristics (gender, age, smoking status, BMI, comorbidity) were included as fixed effects. The continuous predictors age and BMI were centred by subtracting from age 68 years (median age in the sample) and from BMI 25 kg/m^2^ (clinical cut-off for overweight; close to median 26 in sample), to allow interpretation of time effects on the outcome if there is interaction of age or BMI with time [18]. All categorical predictors were entered through dummy indicator coding, with as reference categories respectively baseline for time, non-smoker for smoking status, male for gender, and absence of disease for each comorbidity. Interactions of time with each patient characteristic were included in the model. Finally, outcome differences between therapists were included as a random intercept, and outcome differences between patients (as far as unexplained by the predictors) were accommodated by allowing for an unstructured covariance pattern of the repeated measures. The model was subsequently simplified by excluding non-significant interaction effects (α ≥ .05) from the model one-by-one. All available measurements of all patients were analysed without imputation for missing measurements, which is valid under the same assumptions as multiple imputation, and under less restrictive assumptions than complete cases analysis or simple imputation methods [18]. Values of all repeated measures in maximum pain-free walking distance after the first occurrence of the cut-off value ICD = 1600m were reset to missing because of a maximized incline and testing duration of 30 minutes (1600m) in standard physiotherapy practice. Outcome measures were checked for normality. Predictors were checked for outliers (summary descriptive statistics and histograms) and multicollinearity by inspecting their Variance Inflation Factor (VIF) [19]. No VIFs above 10 were found, indicating absence of multicollinearity [20]. Further, in view of the skewed distribution of the outcome, two additional analyses to check the robustness of the results were performed. First, BMI was left out of the analysis (30% missing values), leading to the inclusion of 1254 more patients into the analysis. Third and last, the outcome was dichotomized as 1 if ICD = 1600m and as 0 if ICD < 1600m, and was analysed with mixed logistic regression using the same modelling procedure as in the mixed linear regression of the continuous ICD measure. The results of the additional analyses were compared with the first analysis. In view of multiple testing, significant results are presented at the 1% and the 5% significance level, and results significant at the 5% level but not at the 1% level must be treated as tentative pending replication.

## Results

### Descriptive data of participants

The inclusion criteria were met by 2995 patients. The average age of patients was 67 years. Comorbidities that were present in more than half of all patients were vascular comorbidity (62%), internal comorbidity (54%) and cardiac comorbidity (49%). Mean initial claudication distance (ICD) at baseline was 270m and after 12 months of SET 667m. The characteristics of the study population are described in Table 1.

**Table 1:** Characteristics of the study population.

Characteristic^a^	Total population	Males	Females
	*N* = 2995^b^	*N* = 1864	*N* = 1131
Age, years	67.4 ± 9.9	67.0 ± 9.5	68.2 ± 10.4
BMI, kg/m^2^	26.8 ± 4.3	26.8 ± 4.0	26.6 ± 4.7
Current smokers	1140 (41.5)	708 (41.5)	432 (41.5)
Orthopaedic comorbidity	570 (23.8)	325 (21.9)	245 (26.9)
Neurological comorbidity	555 (23.6)	353 (24.2)	202 (22.6)
Cardiac comorbidity	1163 (48.7)	756 (50.8)	407 (45.2)
Vascular comorbidity	1474 (61.6)	909 (61.0)	565 (62.5)
Pulmonary comorbidity	408 (17.9)	270 (19.1)	138 (16.0)
Internal comorbidity	1242 (54.4)	755 (53.2)	487 (56.4)
Baseline ICD, meter	269.6 ± 227.3	286.1 ± 235.8	242.8 ± 210.1
1 months ICD, meter	433.6 ± 333.4	456.4 ± 348.7	394.5 ± 301.5
3 months ICD, meter	531.9 ± 380.9	554.5 ±394.4	496.0 ± 355.6
6 months ICD, meter	605.3 ± 411.6	632.9 ± 420.0	562.8 ± 394.9
12 months ICD, meter	667.1 ± 446.1	672.8 ±448.1	658.2 ± 443.3

BMI: Body-Mass Index; ICD = Initial Claudication Distance

^a^Dichotomous variables are presented as N (%) and continuous variables as mean ± standard deviation; ICD is reported as median ± standard deviation.

^b^*N* = 2995 is the maximum amount of patients that were included in one of our analysis.

The results of the three-level mixed linear regression analysis showed at a 1% significance level that being female (p = 0.000), advanced age (p = 0.000) and a high BMI (p = 0.000) lead to less improvement in ICD after SET. Cardiac comorbidity seemed to be associated with improvement in ICD at a 5% level (p = 0.011). In addition, all three interaction terms, time by age (p = 0.000), time by vascular comorbidity (p = 0.023) and time by BMI (p = 0.023) were significantly associated with ICD progress (Table 2). Interactions gender*time and smoking*time were tested but were not significant.

**Table 2:** Final mixed linear model.

Fixed- effects [*N* = 1741]	Estimate	SE	t-value	p-value	95% Confidence Interval	
					Lower	Upper
Intercept	315.48	22.869	13.80	0.000	270.59	360.37
Gender	-40.72	11.613	-3.51	0.000	-63.50	-17.94
Age^a^	-4.06	0.601	-6.75	0.000	-5.24	-2.88
BMI^a^	-4.68	1.338	-3.50	0.000	-7.30	-2.06
Past smoker^b^	19.06	19.109	1.00	0.319	-18.43	56.54
Current smoker^b^	-34.27	19.930	-1.72	0.086	-73.36	4.82
Orthopaedic comorbidity	5.32	13.585	0.39	0.695	-21.32	31.97
Neurological comorbidity	-4.17	13.483	-0.31	0.757	-30.61	22.27
Cardiac comorbidity	-29.55	11.549	-2.56	0.011	-52.20	-6.90
Vascular comorbidity	-11.00	11.288	-0.98	0.330	-33.15	11.14
Pulmonary comorbidity	-17.94	14.875	-1.21	0.228	-47.12	11.24
Internal comorbidity	-9.14	11.708	-0.78	0.435	-32.11	13.82
Time 1 dummy	165.95	7.527	22.05	0.000	151.19	180.71
Time 3 dummy	305.16	11.238	27.15	0.000	283.12	327.21
Time 6 dummy	439.01	18.214	24.10	0.000	403.27	474.75
Time 12 dummy	584.84	29.593	19.76	0.000	526.72	642.97
Age*time	-0.63	0.158	-3.98	0.000	-0.94	-0.32
BMI*time	-0.82	0.359	-2.28	0.023	-1.52	-0.11
Vascular comorbidity*time	-7.07	3.102	-2.28	0.023	-13.17	-0.98

Dependent Variable: ICD (Initial Claudication Distance).

For females the predicted ICD is 41m less than for males after 12 months of SET. For patients with a cardiac comorbidity, the predicted ICD is 30m less after 12 months compared to patients without a cardiac comorbidity. Both interpretations apply when all other variables are kept constant.

To calculate the improvement in ICD from baseline to IC at 1, 3, 6 and 12 months for the interactions with time the following regression equation was used:

$${\text{Change in ICD}}_{1,3,6,12\text{months}}=\text{B}1*\text{t}(1,3,6,12)\text{dum}+\text{b}2*\text{time}*\text{age}(\text{centered})+\text{b}3*\text{time}*\text{BMI}(\text{centered})\;+\text{b}4*\text{time}*\text{vascularcomorbidity}.$$

In general terms, per year increase in age (range 33–93) ICD improvement is 8 meters less after 12 months. Per unit increase of BMI (range: 16–46) the improvement of ICD is 10 meters less after 12 months and the presence of vascular comorbidity leads to 85 meters less improvement after 12 months of SET. In all three scenarios all other variables are hold constant. Fig A, Fig B and Fig C in S2 File provide plots of predicted values for the total sample per subgroup of age, BMI and vascular comorbidity to visualize interactions with time.

To check the robustness of our findings two additional analysis were performed. First, we eliminated BMI from the model (30% missing values), resulting in 1254 more patients in the analysis. The results of this analysis were in agreement with the results of the mixed linear model described in Table 2. Second, in view of non-normality of ICD the analysis was repeated by using a mixed logistic regression model after dichotomizing ICD as 1 if ICD = 1600m and as 0 if ICD <1600m. In accordance with the linear analysis, the results showed that being female (p = 0.000), advanced age (p = 0.000) and a high BMI (p = 0.000) were associated with shorter ICD at all-time points. Furthermore, for current smoking (p = 0.031) and vascular comorbidity there seemed to be a negative association with ICD (p = 0.006). None of the interaction terms were significant, probably due to loss of power by dichotomization.

## Discussion

This study showed that female gender, advanced age, a high BMI and cardiac comorbidity are associated with less improvement in Initial Claudication Distances (ICD) after 1, 3, 6 and 12 months of Supervised Exercise Therapy (SET). In addition, the interaction time by age, time by BMI and time by vascular comorbidity were significantly associated with ICD. Additional analysis showed that there might be a negative association between vascular comorbidity and ICD and well as between cigarette smoking and ICD.

Some limitations are worth noting. First, due to the cross-sectional design of this study no causality can be inferred. Second, the absence of a control group and a randomisation procedure prevents interpretation of time effects as treatment effects. Time effects may also have been detected due to spontaneous recovery or treatments patients follow or experience outside the physiotherapy setting. Third, comorbidity was registered at the first visit, hence before the start of SET. Therefore, it was not possible to investigate whether the presence of comorbid diseases changed over time. Fourth, the exact training program for each individual patient was not recorded. In general, the recommendations stated in the guideline Intermittent Claudication (IC) of the Royal Dutch Society for Physiotherapy are used to treat patients with PAD. However, we have no information about whether the general recommendation was adjusted for an individual patient. Therefore, the influence of variance in treatment on ICD after SET could not be investigated.

Noteworthy is the fact that this study highlights a significant association with ICD for age, gender, BMI, and cardiovascular comorbidity at one go. When looking at previous research, the hampering association of all these factors already has been established [5–10]. However, it was spread throughout different studies, each presenting the adverse effect of one or two patient characteristics and/or comorbidity on walking distance in patients with IC. A possible explanation might be that the sample sizes of previous studies were relatively small (maximum *N* = 460 [6]) in comparison with our study (*N* = 2995). Furthermore, for this study patients were not intentionally recruited, but data from EMRs was used, which allowed the research team to investigate which patient characteristics and comorbidity affect ICD in the standard physiotherapy care setting.

The inverse association between anthropometric indices like gender, age and BMI and walking distance has been previously studied. To illustrate, Troosters et al. [21] highlighted that 66% of the variability in walking distance assessed by the six-minute walk test in healthy participants aged 50–85 years is attributable to age, gender and BMI. Applying these finding to patients with IC, a systematic review of Robeer et al. [22] showed that older patients respond less favourably to SET compared to their younger counterparts. Changes in the cardiovascular system that occur with aging might explain why advanced age reduces the improvement in ICD after SET. Alterations in cardiovascular physiology like a reduction in a heart’s peak capacity to pump blood by 5% to 10% per decade might cause a diminished aerobic capacity with increasing age. Furthermore, with advanced age, the elasticity of arteries decreases and vascular walls thicken. This results in a slower exchange between oxygen and waste products [23]. As intermittent claudication is already associated with a decreased blood flow to the lower extremities particular during times of increased metabolic demand such as during SET, above mentioned age-related cardiovascular adaptations might worsen muscle oxygen supply. Thus, the combination of PAD and age-related cardiovascular adaptations might cause oxygen ischemia faster; the muscle may not receive as much oxygen as needed to support physical activity and this might explain why ICD is lower in older patients with IC compared to their younger counterparts. The capacity to perform aerobic exercise also depends on the ability of the heart to augment its output to the exercising muscles. This ability is often impaired in patients with cardiac comorbidity. Results of the present study show that patients with cardiac comorbidity show fewer improvements in ICD after SET, which might be caused by inadequate blood flow to the active skeletal muscle secondary to impaired cardiac output. Research has shown that patients with cardiac diseases may achieve less than 50% of the maximum cardiac output attained by individuals without cardiac disease [24].

Regarding the inverse association between a high BMI and less improvement in ICD after SET, previous research reported that BMI is a predictor of walking ability and functional capacity. Overall, it seems that increased body mass, either around the hip and thighs (gynoid obesity) or around the belly (android obesity) reduces walking ability and has a negative impact on functional capacity. The results of studies whether high fat mass or low lean mass are more important in the association between obesity and walking capacity remain contradictory. A study by Jankowski et al. [25] and a study by Davison et al. [26] found that a higher fat-mass is more important than low lean mass in the association with mobility-related disability. In contrast, a study of Newman et al. [24] showed an independent effect of low lean mass on walking ability.

The results of the present study also report that females show less improvement in ICD compared to males. Microvascular gender differences in patients with IC have been previously reported and might explain the differential improvement in ICD between men and women [27,28]. A lower haemoglobin oxygen saturation of the calf muscles combined with a more impaired microvascular function seems to cause greater muscle ischemia during SET in women compared to men [27,28]. Other general gender-based biochemical differences like higher levels of oxidative stress and inflammation that are common in postmenopausal women might also contribute to lower improvements in ICD [27]. Nevertheless, other differences such as motivation, willingness to walk through claudication pain and pain tolerance threshold might be additional explanatory factors.

To check the robustness of our findings among others mixed logistic regression analysis was performed. This analysis was the only supplementary analysis reporting that addionally current smoking and vascular comorbidity seemed to be associated with less improvement in ICD after SET. Nevertheless, previous research reported that current smoking is associated with poorer measures of exercise capacity during treadmill testing, including lower peak oxygen update and earlier onset of claudication pain during walking. A study of Afaq et al. [29] found that current smokers have significantly lower rates of tissue oxygenation at peak claudication distance, which might explain the lower ICD improvements in current smokers compared to non- or past-smokers found in this additional analysis. Peripheral factors that impact walking ability and therefore might explain lower ICD due to vascular comorbidity may include abnormalities in the endothelial function like vasodilatory capacity during physical activity and distribution of cardiac output. Since only one of the additional analysis showed current smoking and vascular comorbidity being predictors of ICD, these two findings should be interpreted with caution. Due to dichotomization of the outcome variable we lost power in the analysis, which might also explain why none of the interaction terms was significant.

In conclusion, the present study showed that being female, advanced age, high BMI and cardiac comorbidity are associated with less improvement in ICD after SET. Because this large prospective usual care cohort study showed that the association of all these factors remained in place, subgroups of patients who benefit less from SET have been most certainly identified. Taking care of these patients who benefit less from SET is a crucial preparatory step to be able to early adjust treatment expectations and treatment programme.

## Supporting Information

S1 File Comorbidity. (DOCX)

S2 File Plots of predicted values for the total sample per subgroup of age, BMI and vascular comorbidity to visualise interactions with time. Plot of predicted values for the total sample per subgroup of age **(Fig A).** Plot of predicted values for the total sample per subgroup of BMI **(Fig B).** Plot of predicted values for the total sample per subgroup of vascular comorbidity **(Fig C).**
(PDF)
