# Supplementary material for: Patient Characteristics and Comorbidities Influence Walking Distances in Symptomatic Peripheral Arterial Disease: A Large One-Year Physiotherapy Cohort Study
Source: PLoS One. 2016 Jan 11;11(1):e0146828. doi: 10.1371/journal.pone.0146828 (PMC4708998; doi:10.1371/journal.pone.0146828)
Supplement: S1 File — (DOCX) [file pone.0146828.s001.docx]

**Comorbidity**

**Orthopaedic disease** was considered to be present in patients with one of the following conditions: arthritis, rheumatoid arthritis, polyarthritis, osteoporosis, joint prosthesis, arthrogenic impairment, congenital dysplasia, congenital scoliosis, M. Scheuerman, M. Bechterew, fractures, hyperplasia / malignancy, Reiter's syndrome, Systemic Lupus Erythematous (SLE), psoriasis, polymyositis and other orthopedic disorders. Patients with a cerebrovascular accident / a transient ischaemic attack / central paresis, peripheral nerve disease, cerebellar disorders / encephalopathy, multiple sclerosis / amyotrophic lateral sclerosis / spinal cachexia, Parkinson / Extrapyramidal disorders, hernia nuclei pulposi with motor loss, radiculopathy / radicular syndrome, Paraplegics (traumatic / partial), neurotrauma, hyperplasia / malignancy or other **neurological disorders** in their medical history were considered to have neurological disease. **Cardiac disease** was viewed as the presence of at least one of the following: hypertension, abnormal electrocardiography, abnormal sinus rhythm, abnormal functional capacity, angina pectoris, heart failure, myocardial infarction, coronary arterial bypass graft, percutaneous transluminal coronary angioplasty, rhythm disorders, cardiac decompensation, heart valve suffering and other cardiac pathologies. **Vascular disease** was denoted to be present in patients with one of the following conditions in their medical history: PTA, recanalization, bypass operation, endarterectomy, hypertension, arrhythmia, cardiac decompensation, heart valve suffering and other vascular pathologies. **Pulmonic disease** was defined as the presence of at least one of the following: asthma, interstitial lung disease, diffuse interstitial lung disease / sarcoidosis, emphysema, cystic fibrosis, sleep apnea, hyperplasia / malignancy, chronic obstructive pulmonary disease and other pulmonic disorders. Patients with hypercholesterolemia, hyperlipidemia, elevated homocysteine ​​levels, abnormal lipid spectrum, renal impairment, obesity, hyperplasia / malignancy, immunity disorder, diabetes mellitus and other internal pathologies in their medical history were esteemed to have **internal disease**.
