# Supplementary material for: Patient Characteristics and Comorbidities Influence Walking Distances in Symptomatic Peripheral Arterial Disease: A Large One-Year Physiotherapy Cohort Study
Source: PLoS One. 2016 Jan 11;11(1):e0146828. doi: 10.1371/journal.pone.0146828 (PMC4708998; doi:10.1371/journal.pone.0146828)
Supplement: S2 File — Plot of predicted values for the total sample per subgroup of age (Fig A). Plot of predicted values for the total sample per subgroup of BMI (Fig B). Plot of predicted values for the total sample per subgroup of vascular comorbidity (Fig C). (PDF) [file pone.0146828.s002.pdf]

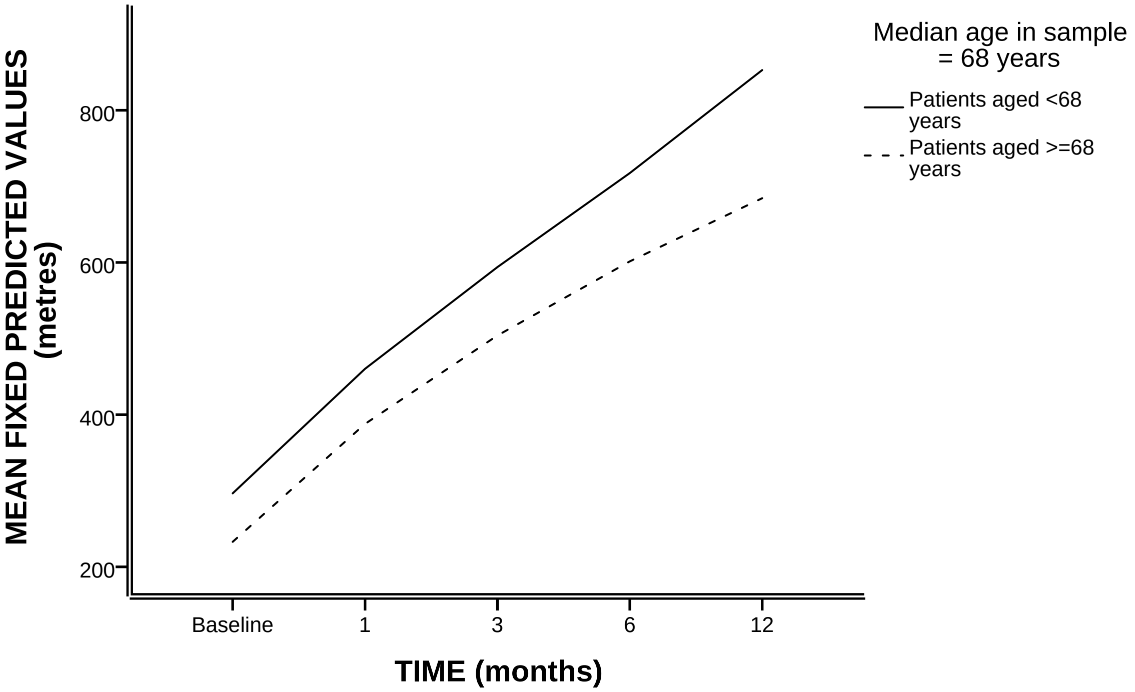

Figure A. Plot of predicted values for the total sample per subgroup of age. Change = B1 + B2*time*agecentered if vascular comorbidity absent and BMI =25.

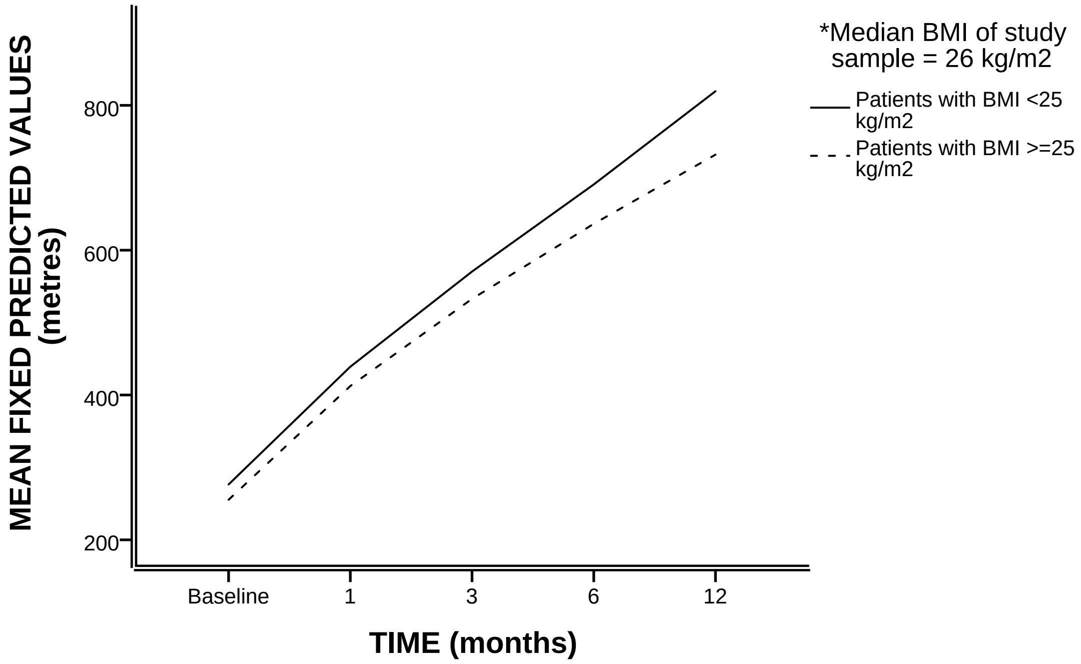

Figure B. Plot of predicted values for the total sample per subgroup of BMI. Change = B1 + B3*time*BMIcentered if age =68 and vascular comorbidity absent.

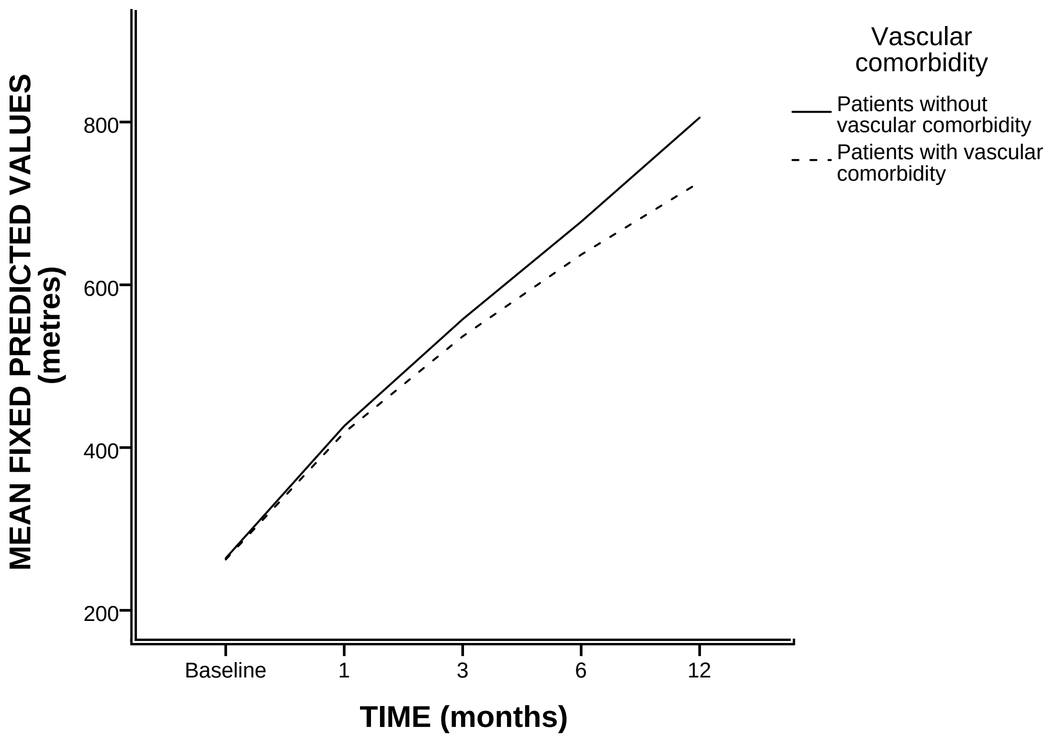

Figure C. Plot of predicted values for the total sample per subgroup of vascular comorbidity. Change = B1 + B4*time*vascular comorbidity if age = 68 and BMI =25.
